# Chemokine Receptor-5Δ32 Mutation is No Risk Factor for Ischemic-Type Biliary Lesion in Liver Transplantation

**DOI:** 10.1155/2009/436515

**Published:** 2009-03-30

**Authors:** Christoph Heidenhain, Gero Puhl, Christian Moench, Anja Lautem, Peter Neuhaus

**Affiliations:** ^1^Department of General, Visceral and Transplantation Surgery, Charité-University Medicine of Berlin, Campus Virchow Klinikum, Augustenburger Platz 1, 13353 Berlin, Germany; ^2^Department of General and Visceral Surgery, J.W. Goethe University Frankfurt/Main, Theodor Stern Kai 7, 60590 Frankfurt/Main, Germany; ^3^Department of Transplantation Surgery, Johannes Gutenberg University of Mainz, Langenbeckstraße 1, 55131 Mainz, Germany

## Abstract

It has been shown that certain chemokine receptor polymorphisms may correspond to certain complications after organ transplantation. Ischemic-type biliary lesion (ITBL) encounters for major morbidity and mortality in liver transplant recipients. So far, the exact cause for ITBL remains unclear. Certain risk factors for the development of ITBL like donor age and cold ischemic time are well described. In a previous study, a 32-nucleotide deletion of the chemokine receptor-5Δ32 (CCR-5Δ32) was strongly associated with the incidence of ITBL in adult liver transplantation. This study re-evaluates the association of CCR-5Δ32 gene polymorphism and the incidence of ITBL. 169 patients were included into this retrospective analysis. 134 patients were homozygous for wild-type CCR-5, 33 patients heterozygous, and 2 patients were homozygous for CCR-5Δ32 mutation. There were no major differences in donor or recipients demographics. No association was found between CCR-5Δ32 mutation and the development of ITBL. We conclude that CCR-5Δ32 is no risk factor for the development of ITBL in our patient cohort.

## 1. Introduction

**Figure 1:**
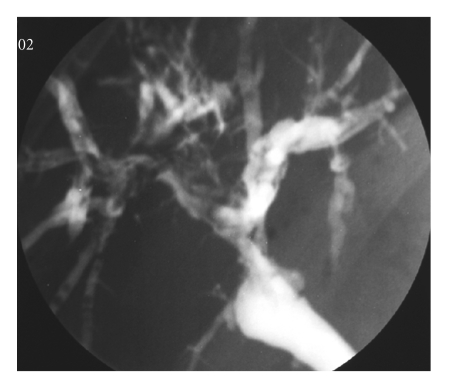
Intrahepatic presentation of ischemic-type biliary lesion six months after hepatic transplantation for chronic hepatitis B-associated liver cirrhosis. The patient's hepatic artery is patent, and there is no other known cause for the destruction of the intrahepatic biliary tract.

The terms “nonanastomotic biliary strictures”, “intrahepatic biliary strictures”, or “ischemic-type biliary lesion” (ITBL) are often used as synonyms for hilar or intrahepatic diffuse bile duct strictures, necrosis, ecstasies, or dilatations (see Figure 1) [1, 2]. The reported incidence of ITBL after OLT varies between 1.4% and 20% [3–5]. Some centers report even higher incidence [6]. Patient and graft survival after the diagnosis of ITBL are significantly reduced [7]. ITBL is the third most common reason for hepatic retransplantation [8]. This complication encounters for major morbidity and mortality, creates high costs, and aggravates organ shortage [7, 8].

The exact cause of ITBL still remains unclear. Only relevant risk factors are described. However, data about risk factors for the development of ITBL are inconsistent. A recent study on 1113 liver transplant patients showed no relevant donor or recipient risk factor of ITBL [5]. There are only two studies evaluating the impact of chemokine receptors (CCR) on the development of ITBL [6, 9]. In Moench's study on 146 OLT patients CCR-5Δ32 mutation was evaluated and correlated with a significant increased incidence of ITBL [6]. A recent study on 137 pediatric liver transplants failed to show an association between CCR-5Δ32 and biliary complications [9]. CCR-5Δ32 is a single base-pair deletion of CCR-5 that leads to a nonfunctional receptor [10]. The clinical impact of this mutation was first described for homozygous CCR-5Δ32 Caucasians being highly resistant to HIV-1 infection [11]. If there was an immunological cause for ITBL, a nonfunctional CCR might be relevant for this complication. Homozygous CCR-5Δ32 patients showed a significant increased renal allograft survival [12]. Experimental studies correlated a nonfunctional CCR-5 with less acute rejection episodes in lung [13], heart [14] and islet cell transplantation [15].

The aim of this study was to re-examine a correlation of CCR-5Δ32 genotype with the susceptibility of ITBL within our patients.

## 2. Patients and Methods

169 liver transplant patients were analyzed retrospectively. All patients were transplanted at the transplant center of the Humboldt University of Berlin between 03/2002 and 03/2005 and were included during routine Follow-up examination. Follow-up period was 24 months minimum. 11 patients with the established diagnosis of ITBL, that were transplanted earlier than 03/2001, were selectively included into this study due to the low incidence of ITBL of only 4.0% within our patients. The diagnosis of ITBL was made within the first year after transplantation in 82% of the patients. The following demographic data were extracted from the hospital records and evaluated: age, gender, underlying liver disease, blood group, Child-Pugh score (CPS), model for end stage liver disease score (MELD score), initial immunosuppression, cytomegalovirus infection (CMV), HLA match, donor age and gender, donor serum sodium, cause of brain death and length of stay on intensive care unit (ICU) prior to organ harvesting. 154 patients received a cadaver graft, 15 patients received a graft from a living donor. Altogether, 19 split-liver transplantation were included. The local ethic committee approved the study. Written informed consent was obtained from all patients before blood was taken for DNA analysis.

## 3. Definition of ITBL

ITBL was defined as nonanastomotic intra- or extrahepatic biliary strictures without any history of hepatic artery complications, ABO, incompatibility or other known causes of bile duct damages. In all cases patency of the hepatic artery was proved by Doppler ultrasound, computer tomography based angiography or conventional angiography. Recurrence of primary biliary cirrhosis (PBC) or primary sclerosing cholangitis (PSC) and vanishing bile duct syndrome were ruled out in all cases by liver biopsy. Diagnosis of ITBL was always established with endoscopic retrograde cholangiography or percutaneous transhepatic cholangiography.

## 4. Genotype Analysis

All genotype analyses were performed at the Johannes Gutenberg University of Mainz, Department of Transplantation Surgery. For analysis of the CCR-5 genotype, genomic DNA was prepared from 200 *μ*L peripheral blood using the QIAamp DNA blood kit (Qiagen, Cologne, Germany). 2.5 *μ*L of DNA were amplified by PCR using the following CCR-5 specific primers: CCR-sense, 5′-CAAAAAGAAGGTCTTCATTACACC-3′ and CCR-5-antisense, 5′-CCTGTGCCTCTTCTTCTCATTTCG-3′. The PCR mixture was composed of 2.5 *μ*L 10 x PCR buffer (Roche Molecular Systems, Mannheim, Germany), 0.5 *μ*L of 12.5 mmol/L dNTP (PeqLab, Erlangen, Germany), 2.5 *μ*L of each sense and antisense primer (10 *μ*mol/L), and 1.25 U AmpliTag DNA polymerase (Roche Molecular Systems) in a total volume of 25 *μ*L. Forty PCR cycles were run on a Genius thermocycler (Techne, Cambridge, UK), using the following temperature profile: initial denaturation, 94°C 3 minutes; amplification, 94°C 1 minute, 64°C 1 minute, and, 72°C 1 minute (40 cycles); terminal elongation, 72°C 9 minutes. The size of the wild-type product was 189 base pairs (bp), and the CCR-5Δ32 allele yielded a product of 157 bp. PCR products were analyzed by 2% agarose gel electrophoresis.

## 5. Statistical Analysis

All statistical calculations were performed in SPSS 11.3 (SPSS Inc., Chicago, USA). Data are given as mean values ± standard deviation. Descriptive statistics were used to summarize the donor and recipients characteristics. For independent variables, cross tabulations and chi-square tests were performed. Nonparametric variables were evaluated with Fisher's exact test, and asymptotic significance was calculated.

All of the tests performed were two-sided. *P*-values of *P* < .05 were considered as statistically significant. All calculations were performed in association with the Department of Biometrical Medicine of the Humboldt University of Berlin.

## 6. Results

### 6.1. Patient Characteristics and Genotype Distribution

A total number of 169 liver transplant recipients were available for genotyping and complete data analysis. Gender and age were equally distributed between wild-type group (wt/wt) and heterozygous CCR-5Δ32 group (wt/Δ32). Patients in the homozygous group (Δ32/Δ32) were female and male. The observed genotype frequency was as expected assessed by Hardy-Weinberg equilibrium in the study population. There were no differences between wt/wt group and wt/Δ32 group regarding to CPS score, MELD score or blood group (Table 1).

There were no statistical significant differences in the composition of underlying liver disease of group wt/wt and wt/Δ32. Both patients with Δ32/Δ32 had primary biliary cirrhosis as underlying liver disease.

Initial immunosuppression was tacrolimus based in 82.6% in the wt/wt group compared to 84.8% in the wt/Δ32 group. Likewise, cold ischemic time and HLA match showed no differences between groups. Both homozygous Δ32 patients had zero HLA match. CMV infection that demanded ganciclovir treatment was present in approximately 30% in the wt/wt and wt/Δ32 group and in both homozygous patients.

### 6.2. Donor Characteristics

There were no differences between group regarding donor age or gender. Donors of group Δ32/Δ32 were younger (35.7 years versus 46.5 years and 48.5 years). Mean donor serum sodium was 146.9 mmol/L in the wt/wt group compared with 147.7 mmol/L in the wt/Δ32 group and 155.5 mmol/L in the Δ32/Δ32 group. Data of causes of brain death and length of stay on the ICU prior to organ harvesting are shown in Table 2.

### 6.3. Incidence ITBL and Rate of Retransplantation

Incidence of ITBL was 11.2% in this study due to the selection of patients with ITBL that were additionally included into this evaluation. Homozygous Δ32 patients developed no ITBL compared to 11.2% and 12.1% of homozygous wild-type patients and heterozygous patients, respectively. The rate of retransplantation was 3.0% in both wt/wt and wt/Δ32 group (see Table 3). Retransplantation of the heterozygous patient was indicated due to chronic ductopenic rejection following OLT for PSC. In the wt/wt group, the indications for retransplantation were INF, cryptogenic recirrhosis, and ITBL.

## 7. Discussion

The problem of genetic association studies and complex clinical syndromes or diseases must be addressed. One can always question the usefulness of these studies that are often even small in sample size. Most of these studies are statistically underpowered. On the other side, it seems important to undertake replication studies for reported associations between genetic polymorphisms and diseases, especially in diseases of major clinical importance.

In this study, the distribution of heterozygous Δ32 and homozygous Δ32 mutation was very consistent with the published data of the global distribution of this gene polymorphism [10, 16]. Heterozygous and homozygous genotypes occur in Caucasian population in 15%–20% and 1%, respectively [16]. Heterozygous individuals show no abnormal receptor function compared with wt/wt individuals. All examined donor and recipient factors showed no statistical differences between groups. This seems important due to the small number of patients included in this study and the possible bias by including selected patients with the diagnosis of ITBL into the study cohort.

Despite increasing success rates in clinical OLT over the past decades, ITBL remains a major cause for recipient morbidity and mortality [1–5]. This single complication creates enormous costs and aggravates organ shortage. Up till today, only risk factors for ITBL could be identified in various clinical studies. The length of cold ischemic time was correlated with the development of ITBL [1–4]. Donor age was found to be a significant risk factor for ITBL. Other studies were not able to show these correlations [5]. Immunological causes seem to play only a minor role in the pathogenesis of ITBL. Moench et al. described a single base-pair deletion in the coding region of the chemokine receptor-5Δ32, CCR-5Δ32, to be a significant risk factor for the development of ITBL. In Moench's study on 146 OLT patients CCR-5Δ32 was a significant risk factor for ITBL (incidence of ITBL in CCR-5Δ32 patients was 30% versus 11.7% in CCR-5 wild-type patients) and was correlated with a decreased survival rate after OLT. The overall ITBL rate was 15% [6]. The different incidence of ITBL of the study by Moench and this study may be a reason for the different findings, even though both investigators used the same definition of ITBL. Donors were younger in this study with 38.2 ± 16 (non-ITBL patients) and 42.9 ± 17 (ITBL) versus 46 ± 14 (non-ITBL) and 52 ± 14 (ITBL) in Moench's study. However, cold ischemic time was shorter in Moench's investigation (564 minutes (ITBL) and 538 minutes (non-ITBL) versus 637 minutes (ITBL) and 558 minutes (non-ITBL)). The use of arterial back table perfusion was also routinely done for all organs that were harvested by a team of our own. Fischer-Maas et al. analyzed CCR-5Δ32 polymorphism in 137 pediatric patients but showed no correlation with biliary complications [9]. The incidence of ITBL varies between 1.4% and 20% according to the literature, which might be a problem of different definition of this disease [3–5]. The rate of ITBL in our OLT patients (2100 patients between 1988 and 2004) is 4.0%. In the presented study on 169 OLT patients the overall incidence of ITBL was 11.7%, but only due to a selective inclusion of patients with the established diagnosis of ITBL. This practice of patient recruitment may be criticized, but we think it is justified according to our low incidence of ITBL. Thus, it was possible to investigate 19 patients with ITBL. ITBL rate in CCR-5Δ32 patients was virtually equal to the one in CCR-5 wild-type patients. No statistically significant differences regarding ITBL or retransplantation were observed.

Why would CCR-5Δ32 mutation promote the development of ITBL? CCR-5Δ32 is a 32-base-pair deletion within the coding region of CCR-5, which results in a frame shift and generates a nonfunctional receptor [11]. Homozygous expression of CCR-5Δ32 is associated with a reduced risk of asthma and with a reduced severity of rheumatoid arthritis [17, 18], multiple sclerosis [19, 20], and primary biliary cirrhosis (PBC) [21]. In other words, the nonfunctional nature of CCR-5Δ32 protects the individual from autoimmune diseases where CCR-5 seems to play a central pathophysiological role. These data do not backup the theory, that immunological risk factors are dominant in the development of ITBL. Likewise, a correlation of a reduced survival rate with CCR-5Δ32 would not be consistent to the literature, where CCR-5Δ32 mutation is associated with an increased survival in renal [12], lung [13], heart [14] and islet cell transplantation [15]. In contrast to those findings, CCR-5Δ32 is strongly associated with an increased severity of PSC [22]. Patients suffering from PSC have been described as carrying a higher risk for ITBL, with a reported significantly increased incidence of 15.8% to 25% [1, 8, 23]. Another study reported PSC as the only independent risk factor for ITBL with an incidence of 31% compared with 9% of the control group [24]. However, the problem of differentiation between ITBL and recurrence of PSC must be addressed. Recurrence rates of 8.6% to 25% were described for PSC after OLT [25–27]. The diagnose of recurrence of PSC is based on cholangiographic findings of intrahepatic, hilar and/or exrahepatic strictures, duct irregularities and on the histopathological picture of fibrous cholangitis and/or fibro-obliterative lesions with or without ductopenia, biliary fibrosis, or biliary cirrhosis [28]. Most of these findings are neither pathognomonic for either recurrence of PSC nor ITBL [29]. All patients with ITBL in this study underwent percutaneous liver biopsy, and our pathologists ruled out PSC recurrence. There remains a diagnostic uncertainty.

Since two of three studies failed to show an association between ITBL and CCR-5Δ32 gene polymorphism, a general recommendation for screening of OLT patients for CCR-5Δ32 does not seem to be justified.

## Figures and Tables

**Table 1:** Recipient characteristics. MELD: model for end-stage liver disease; CPS: Child-Pugh score; BG: recipients blood group; wt/wt: wild-type CCR-5; wt/Δ32: heterozygous CCR-5Δ32; Δ32/Δ32: homozygous CCR-5Δ32; CMV: cytomegalovirus; OKT-3: monoclonal murine anti-CD-3 antibody.

Recipient characteristics				
Variables	wt/wt	wt/Δ32	Δ32/Δ32	*P*-value
*n* (169)	134 (79.3%)	33 (19.5%)	2 (1.2%)	
Recipient gender				
Male	89 (66.4%)	22 (66.7%)	—	.137
Female	45 (33.6%)	11 (33.3%)	2 (100%)	
Mean recipient age (years)	50.2 ± 10.3	49.8 ± 9.9	47.5 ± 17.7	.443
MELD Score (mean ± SD)	17.2 ± 8.7	17.9 ± 9	—	.960
CPS A	13 (11.8%)	4 (16%)	1 (50%)	.487
CPS B	60 (54.5%)	13 (52%)	1 (50%)	
CPS C	37 (33.7%)	8 (32%)	—	
BG A	52 (39.1%)	10 (30.3%)	1 (50%)	.823
BG B	19 (14.3%)	4 (12.1%)	—	
BG AB	15 (11.3%)	3 (9.1%)	—	
BG O	47 (35.3%)	16 (48.5%)	1 (50%)	
Hepatitis B-related cirrhosis	16 (11.9%)	3 (9.1%)	—	.918
Hepatitis C-related cirrhosis	21 (15.7%)	6 (18.2%)	—	
Hepatocellular carcinoma	19 (14.2%)	4 (12.1%)	—	
Primary biliary cirrhosis	6 (4.5%)	—	2 (100%)	
Primary sclerosing cholangitis	4 (3%)	2 (6.1%)	—	
Acute liver failure	4 (3%)	3 (9.1%)	—	
Autoimmune hepatitis	2 (1.5%)	—	—	
Metabolic liver diseases	2 (1.5%)	3 (9.1%)	—	
Alcohol-induced cirrhosis	41 (30.6%)	9 (27.3%)	—	
Retransplantation	4 (3%)	1 (3%)	—	
Others	15 (11.2%)	2 (6.1%)	—	
Cold ischemic time (minutes)	533 ± 144	582 ± 202	633	.806
Initial Immunosuppression				
Tacrolimus	110 (82.6%)	28 (84.8%)	2 (100%)	.824
Cyclosporine A	23 (17.2%)	4 (12.1%)	—	
others	1 (0.7%)	1 (3.0%)	—	
HLA match				
0 match	24 (26.4%)	6 (27.3%)	2 (100%)	.448
1 match	40 (44.0%)	8 (36.4%)	—	
2 matches	21 (23.1%)	5 (19.2%)	—	
3 matches	5 (5.5%)	2 (9.1%)	—	
4–6 matches	1 (1.1%)	1 (4.5%)	—	
CMV Infection				
postitve	43 (32.1%)	12 (36.4%)	2 (100%)	.117
negative	28 (20.9%)	2 (6.1%)	—	
unknown	63 (47.0%)	19 (57.6%)	—	

**Table 2:** Donor characteristics. ICU: intensive care unit; wt/wt: wild-type CCR-5; wt/Δ32: heterozygous CCR-5Δ32; Δ32/Δ32: homozygous CCR-5Δ32.

Donor characteristics				
	wt	wt/Δ32	Δ32/Δ32	*P*-value
Donor age (years)	46.5 ± 17.2	48.5 ± 16.5	35.7 ± 11.2	.663
Donor gender
Male	76 (56.7%)	20 (60.6%)	1 (50%)	.901
Female	58 (43.3%)	13 (39.4%)	1 (50%)	
Mean donor serum Na^+^ (mmol/L)	146.9 ± 8.4	147.7 ± 7.9	155,5 ± 27.5	.552
Cause of brain death				
Subarachnoidal bleeding	75 (56%)	16 (48.5%)	1 (50%)	.866
Trauma	31 (23.1%)	8 (24.2%)	—	
Intracerebral bleeding	1 (0.7%)	1 (0.6%)	—	
Hypoxia	3 (2.2%)	—	—	
Brain tumor	1 (0.7%)	—	—	
Cardiac infarction	1 (0.7%)	—	—	
Cerebral infarction	10 (7.5%)	3 (9.1%)	—	
others	12 (9%)	5 (15.2%)	1 (50%)	
Stay on the ICU prior to Organ harvesting (days)	4.3 ± 4.7	4.7 ± 3.8	7.0	.564

**Table 3:** Events after transplantation. wt/wt: wild type CCR-5; wt/Δ32: heterozygous CCR-5Δ32; Δ32/Δ32: homozygous CCR-5Δ32; ITBL: ischemic-type biliary lesion; Re-OLT: retransplantation.

Incidence of ITBL or Re-transplantation				
Events	wt/wt	wt/Δ32	Δ32/Δ32	*P*-value
*n*	134	33	2	
No ITBL	119 (88.8%)	29 (87.9%)	2(100%)	.870
ITBL	15 (11.2%)	4 (12.1%)		
No Re-OLTx	130 (97%)	32 (97%)	2 (100%)	.970
Re-OLTx	3(3%)	1 (3%)	—	
